# Interference across time: dissociating short from long temporal interference

**DOI:** 10.3389/fpsyg.2024.1393065

**Published:** 2024-07-24

**Authors:** Ilanit Hochmitz, Ahmad Abu-Akel, Yaffa Yeshurun

**Affiliations:** ^1^The Institute of Information Processing and Decision Making, University of Haifa, Haifa, Israel; ^2^School of Psychological Sciences, University of Haifa, Haifa, Israel; ^3^The Haifa Brain and Behavior Hub (HBB), University of Haifa, Haifa, Israel

**Keywords:** interference, temporal crowding, visual masking, mixture-model analysis, similarity

## Abstract

Our ability to identify an object is often impaired by the presence of preceding and/or succeeding task-irrelevant items. Understanding this temporal interference is critical for any theoretical account of interference across time and for minimizing its detrimental effects. Therefore, we used the same sequences of 3 orientation items, orientation estimation task, and computational models, to examine temporal interference over both short (<150 ms; visual masking) and long (175–475 ms; temporal crowding) intervals. We further examined how inter-item similarity modifies these different instances of temporal interference. Qualitatively different results emerged for interference of different scales. Interference over long intervals mainly degraded the precision of the target encoding while interference over short intervals mainly affected the signal-to-noise ratio. Although both interference instances modulated substitution errors (reporting a wrong item) and were alleviated with dissimilar items, their characteristics were markedly disparate. These findings suggest that different mechanisms mediate temporal interference of different scales.

## 1 Introduction

In everyday life, when we try to identify objects that are relevant to our intended actions, there is often interference generated by irrelevant objects that are nearby (spatial interference) and/or appear before and after the objects of our interest (temporal interference). These interferences virtually affect all everyday tasks and our ability to interact with the environment effectively. Spatial interference was studied extensively including examining whether different instances of spatial interference (e.g., spatial crowding, lateral masking, contour interaction) are mediated by a single or multitude of mechanisms (e.g., Levi et al., 2002; Pelli et al., 2004; Petrov et al., 2007; Pelli, 2008; Yeshurun and Rashal, 2010; Whitney and Levi, 2011; Ester et al., 2014; Rashal and Yeshurun, 2014; Lev and Polat, 2015; Strasburger, 2020). In the temporal domain, interference over short temporal intervals, up to a stimulus-onset-asynchrony (SOA) of 100–150 ms—often referred to as visual masking—is an extensively studied phenomenon associated with the reduction or even elimination of the perception of the task-relevant target (e.g., Breitmeyer, 1984; Gorea, 1987; Breitmeyer and Öğmen, 2000, 2006; Enns and Di Lollo, 2000; Enns, 2004). Recently, temporal interference over long intervals, termed temporal crowding, was also demonstrated (Bonneh et al., 2007; Yeshurun et al., 2015; Tkacz-Domb and Yeshurun, 2017; Tkacz-Domb and Yeshurun, 2021; Sahar and Yeshurun, 2024). Though it shares some similarities with spatial crowding, temporal crowding is a qualitatively different phenomenon. As was recently found by Sahar and Yeshurun (2024), similar to spatial crowding, the interference brought about by temporal crowding is not restricted to the periphery and can also occur with central vision. Yet, unlike spatial crowding, its magnitude does not scale with eccentricity. This latter finding is consistent with findings demonstrating that the two phenomena reflect distinct perceptual mechanisms (Tkacz-Domb and Yeshurun, 2021).

While we know quite a lot about visual masking (henceforth just ‘masking’), our current knowledge of temporal crowding is quite limited, particularly in its ‘pure temporal’ form—when all items appear at the same location with no spatial interactions. We do know that the magnitude of the interference caused by temporal crowding is strongly affected by the temporal interval between the target and the irrelevant distractors; shorter target-distractor intervals (i.e., shorter SOAs) generate stronger interference. Critically, the interference brought about by temporal crowding is observed with SOAs that exceed the typical limits of ordinary masking (i.e., SOA > 150 ms), and was even found with SOAs longer than 400 ms, when there was no temporal uncertainty, or when an attentional cue indicated the relevant location (Yeshurun et al., 2015; Tkacz-Domb and Yeshurun, 2017; Tkacz-Domb and Yeshurun, 2021). Nevertheless, the relations between temporal crowding and masking as well as the extent by which these are indeed two separate phenomena remain unclear. The overarching goal of this study was thus to narrow this knowledge gap. This is important because a better understanding of the processes underlying temporal interference of different scales is a critical step for a comprehensive theory of temporal perception, and it is necessary for any attempt to remedy such interference or alternatively utilize it for further research.

Recently, Tkacz-Domb and Yeshurun (2021) examined which aspects of visual processing are impaired by temporal crowding, using an orientation estimation task combined with a temporal crowding paradigm. Participants viewed a sequence of three randomly oriented items, separated by relatively long SOAs (170–475 ms). The target was the second stimulus. They then had to reproduce the target’s orientation by rotating a probe line. Estimation errors, defined as the difference between the target orientation and the reported orientation, were analyzed using the two-misreport mixture model (Shechter and Yashar, 2021). This model enables the extraction of four parameters: (1) The width (*sd*) of a Gaussian distribution of errors that is centered around the target’s orientation. This parameter reflects the error variance of trials in which the target was encoded to some degree. It conveys the encoding precision or the quality of the target representation; the larger the *sd*, the lower the quality of the target representation; (2) The proportion of trials on which the subject responds at random (*g*). This parameter indicates the guessing rate reflecting the frequency of trials in which the target was not registered at all. This is mainly determined by the signal-to-noise ratio (SNR); the higher the *g*, the lower the SNR (Agaoglu et al., 2015); (3, 4) the rate of substitution errors—mistakenly reporting the orientation of the first (*β_1_*) or the second (*β_2_*) distractor, instead of the target. The *β_1_* and *β_2_* parameters are modeled by additional Gaussian distributions centered on the distractors’ orientation. Analyzing the effects of SOA on these 4 parameters revealed a significant increase in the *sd*, *β_1_*, and *β_2_* parameters with decreasing SOA, but there was no significant SOA effect on the *g* parameter. Thus, temporal crowding degrades the quality of target representation and increases substitution errors with both distractors, but it does not affect the SNR. Critically, this pattern was practically opposite to that found by Agaoglu et al. (2015) who also used an orientation estimation task but with a classical masking paradigm (i.e., SOAs <150 ms). With masking, the SOA mainly affected the SNR but not the *sd*, suggesting that temporal crowding and masking are distinct phenomena. However, the two studies differed in various methodological aspects. In Tkacz-Domb and Yeshurun, the target was a ‘clock-face’ stimulus with 360 possible orientations, there were always 2 distractors, one preceding and another succeeding the target, and they differed from the target only in their orientation. In Agaoglu et al. (2015) the target was a single line segment with 180 possible orientations, there was only a single mask, composed of three oriented lines with a different contrast from the target, and it either preceded or succeeded the target. Given these differences, we cannot tell whether the differential outcomes of these studies indicate unequivocally that masking and temporal crowding are two different phenomena. Thus, to gain a better understanding of temporal interference of different time scales and specifically uncover the relations between masking and temporal crowding, the current study uses the same stimuli and overall procedure to directly compare the pattern of SOA effects generated by these two phenomena.

Another way to compare the interference brought about by temporal crowding and masking is by examining what factors affect the two and in what manner. One such factor is inter-item similarity. Several studies reported that both forward and backward masking are stronger when the target and mask are similar. This pattern was found for orientation, color, spatial frequency, form, and depth (e.g., Bevan et al., 1970; White and Lorber, 1976; Lehmkuhle and Fox, 1980; Oyama et al., 1983; Enns, 2004; Bhardwaj et al., 2012). Often, these effects were explained in terms of perceptual segregation. If the target and mask differ in color, for example, the target segregates more easily from the mask, and masking decreases (e.g., Oyama and Yamada, 1978; Oyama et al., 1983). What about temporal crowding? Tkacz-Domb and Yeshurun (2021) found that when the target and distractors had different luminance—the target was black and the distractors were white—the SOA effects were comparable to when all items were black. Critically, the direct comparison of the similar and dissimilar conditions revealed a ‘dissimilarity benefit’ for both *β_1_* and *β_2_* parameters; substitution errors were less frequent when the target and the distractors were dissimilar. However, here too, there are many methodological differences between the masking and temporal crowding studies. Additionally, masking studies did not examine how inter-item similarity affects different aspects of visual processing. Thus, the primary aim of this study was to examine the relations between temporal crowding and masking. Are these two phenomena mediated by similar processes or do they reflect different processes? Perhaps they are similar in some aspects but differ in others. Unlike previous studies, the current study used the same stimuli and task to measure temporal crowding and masking, both with similar and dissimilar target and non-target stimuli, allowing a direct comparison between the two phenomena.

Our secondary goal was to better understand the processes underlying the dissimilarity benefit in temporal crowding. One possibility is that the dissimilarity benefit rests on the availability of grouping cues at the early stages of visual processing. When a dissimilarity benefit was observed for spatial crowding, it was often attributed to grouping processes; when the target and distractors are similar, they form one perceptual group resulting in impaired performance, but when they are dissimilar, they are segregated into separate units which weakens spatial crowding (e.g., Livne and Sagi, 2007; Sayim et al., 2010; Manassi et al., 2012). As grouping also occurs in the temporal domain it may also account for the dissimilarity benefit found for temporal crowding. However, the fact that Tkacz-Domb and Yeshurun used a fixed, known, target’s luminance that was different from the distractors introduces alternative explanations because it might have encouraged participants to adopt higher-level strategies to improve their performance (e.g., inhibit all white items). Additionally, the significant SOA effects on substitution errors suggest that temporal crowding is partially due to source confusion (i.e., all the items are properly encoded, but sometimes the participants confuse the onset time of each stimulus and end up reporting the orientation of a distractor). It is possible, therefore, that the observers have used the target’s unique feature to reduce source confusion thereby reducing the substitution rate. Thus, to better understand the dissimilarity benefit found for temporal crowding one has to prevent the use of such higher-level strategies.

**Figure 1:**
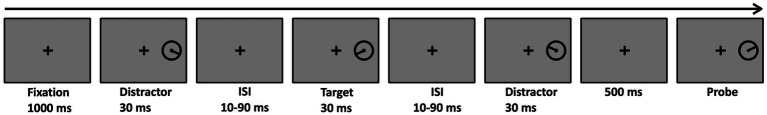
An example of a single trial in the masked condition of Experiment 1. There were five possible target-distractor SOAs (40, 60, 80, 100, and 120 ms). The SOA was fixed within a trial but varied between trials. In the unmasked condition, only the target appeared. The task was to reproduce the target’s orientation.

To achieve these goals, we conducted three experiments. In these experiments, a sequence of 3 randomly oriented items was followed by a probe (Figure 1). The task was to rotate the probe to reproduce the orientation of the second item in the sequence—the target. In Experiment 1, all stimuli were black and the SOAs were chosen to meet the limits of masking (≤120 ms). This allowed us to conduct a more straightforward comparison with temporal crowding. If a different pattern of results arises it will indicate that these are distinct phenomena that rely on different visual processes. Experiments 2 and 3 were designed to compare the effects of similarity on masking vs. temporal crowding. In Experiment 2 the SOAs were relatively long (175–475 ms), and the target and distractors were of different luminance. Importantly, luminance differences varied randomly across trials to ensure the observers did not have advanced knowledge of a target-unique feature. This allowed us to examine the possible involvement of ‘low-level’ effects of similarity (e.g., grouping) on temporal crowding. In Experiment 3, the SOAs were again short, but the target and distractors assumed different luminance that varied across trials allowing us to compare the effects of similarity on masking vs. temporal crowding. If the dissimilarity benefit has different characteristics for masking and temporal crowding this further supports the assertion that these are two separate phenomena.

## 2 Methods

### 2.1 Experiment 1

#### 2.1.1 Participants

Sixteen students from the University of Haifa (8 females, 8 males; age range: 19–34; mean age: 24.9 years) participated in this experiment. All participants provided their informed consent and received course credit or monetary payment of 40 ILS per hour for their participation. The participants were naïve as to the purpose of the experiment and reported normal or corrected-to-normal vision. The sample size choice was based on a power analysis conducted with the R *pwr* package (Champely, 2020). This analysis indicated that 12 participants is the minimum sample size required for the examination of SOA effects with a power of 0.95 and *α* = 0.05. The *F* values, degrees of freedom, and effect sizes used in this analysis were based on Tkacz-Domb and Yeshurun (2021; *F*(4,56) = 6.05, *η*_p_^2^ = 0.30, *N* = 15). This analysis confirmed that the current study sample size had sufficient statistical power. This study adhered to the Declaration of Helsinki and was approved by the ethics committee of the University of Haifa (287/19).

##### 2.1.1.1 Exclusion procedure

An overall-performance score was calculated for each participant (Agaoglu et al., 2015; Sahar and Yeshurun, 2024):


(1) $$\mathrm{Overall}-\mathrm{performance}=1-\left(\mathrm{mean}\;\mathrm{absolute}\;\mathrm{error}/180\right)$$

An overall-performance score around 0.5 indicates that the participant always guessed the target’s orientation because in this case the average of the absolute error should be around 90°. Thus, we excluded participants whose score was below 0.55. None of the participants was excluded in this experiment.

#### 2.1.2 Stimuli and apparatus

Stimuli were presented on a 19″ monitor of an IBM-compatible PC (1,024 × 768 resolution at a refresh rate of 85 Hz), using MATLAB and the Psychophysics Toolbox extensions (Brainard and Vision, 1997; Kleiner et al., 2007). Eye movements were monitored monocularly (right eye) with an EyeLink 1,000 eye tracker (temporal resolution of 1,000 Hz; SR Research, Ottawa, Ontario, Canada). In the masked condition, a sequence of three stimuli was presented to the right or left of a central fixation circle (diameter 0.3°) at an eccentricity of 9° (Figure 1). The target was the second stimulus in the sequence. Each stimulus consisted of a black circle (0.01 cd/m^2^; diameter: 2°) with an inner line (1°). The orientation of the line varied randomly between 360 possible orientations while maintaining the constraint of a different orientation for each stimulus in the sequence. Stimuli were separated by a variable SOA: 40, 60, 80, 100, and 120 ms. The probe also consisted of a black circle and an inner oriented line; its initial orientation was determined randomly. In the baseline (unmasked) condition, a single stimulus, defined as the target, was presented. All stimuli were presented on a uniform gray background (23.5 cd/m^2^).

#### 2.1.3 Procedure

Each trial began with a fixation mark presented for the entire trial. In the masked condition, after 1,000 ms, the three-stimuli sequence was presented, with the target being second in that sequence. Each stimulus was presented for 30 ms. The SOA between the stimuli in the sequence was constant within a trial but varied randomly between trials. In the unmasked condition, only the target appeared. Following the offset of the third stimulus in the sequence (or the single target in the unmasked condition), a blank screen with the fixation mark appeared for 500 ms followed by the probe. The participants reported the target’s orientation by clicking with the mouse on the circle outline, and they could adjust their response for as long as required without a time limit. Once participants reached the desired orientation, they pressed the space bar and the next trial began. The participants had to fixate their gaze on the central fixation until the probe presentation, once the probe appeared, they could move their eyes. Target–distractor SOA was counterbalanced and presented in random order. Altogether, there were 600 experimental trials consisting of 100 trials for each SOA condition and additional 100 trials for the unmasked condition. The experimental trials were preceded by a practice session consisting of 60 trials identical to the experimental trials.

#### 2.1.4 Model fitting

First, we removed from further analysis trials in which a saccade with an amplitude greater than 1° was executed or if the participant pressed the space bar without clicking on the probe. The models’ parameters were estimated using the *MAP* function of MemToolbox developed by Suchow et al. (2013), which finds the maximum *a posteriori* (MAP) parameter estimates. A detailed description of the fitting procedure can be found in Suchow et al. (2013) as well as in the tutorial that accompanies the toolbox – https://visionlab.github.io/MemToolbox.

The two-misreport mixture model was fitted to the error distribution of the masked conditions (separately for each SOA condition of each participant):


(2) $$p\left(\theta \right)=\left(1-g-\beta_{1}-\beta_{2}\right)\varphi_{\sigma }\left(\theta \right)+\frac{g}{2\pi }+\beta_{1}\varphi_{\sigma }\left(\theta_{1}^{*}\right)+\beta_{2}\varphi_{\sigma }\left(\theta_{2}^{*}\right)$$

where *θ* is the orientation error relative to the target (i.e., the difference between the orientation of the target and the reported orientation); *g* reflects the proportion of trials on which the subject responds at random (the guessing rate); *φ_σ_* indicates the circular analog of the Gaussian distribution (the Von Mises distribution) with a mean equal to zero (zero error) and standard deviation *σ (sd)*; *β_1_* is the rate of mistakenly reporting the orientation of the 1st distractor (preceding the target); and *β_2_* is the rate of mistakenly reporting the orientation of the 2nd distractor (succeeding the target). Finally, θ_1_^*^ and θ_2_^*^ are the errors relative to the orientation of the 1st and 2nd distractor, respectively. The *sd* of the Von Mises distribution was assumed to be the same for all stimuli (target and distractors).

In the unmasked condition, only the target was presented (i.e., there were no distractors) and the error distribution of this condition was, therefore, fitted using the standard mixture model that has only 2 free parameters (*g*, *sd*):


(3) $$p\left(\theta \right)=\left(1-g\right)\varphi_{\sigma }\left(\theta \right)+g/2\pi$$

To confirm we were using the optimal model for our data, we compared the two-misreport mixture model (Eq. 2) with the swap model (Bays et al., 2009) that aggregates the contribution of different distractors and, therefore, has only three free parameters – *sd*, *g, β*:


(4) $$p\left(\theta \right)=\left(1-g-\beta \right)\varphi_{\sigma }\left(\theta \right)+g/2\pi +\beta /m\overset{m}{\underset{i}{\sum }}\varphi_{\sigma }\left(\theta_{i}^{*}\right)$$

Where *m* is the number of distractors (2 in this study), and θi* is the error relative to the orientation of the ith distractor. We also compared the two-misreport mixture model and the swap model to versions of these models that include a bias term. These two additional models were similar to the regular two-misreport and swap models except that the mean (*μ*) of the von Mises distribution around the target (*φ_σ_, μ*) was a free parameter. We used the Akaike information criterion with correction (AICc) to compare these models. This criterion includes a penalty term for each additional model parameter. Models’ comparison was performed using MemToolbox (Suchow et al., 2013).

### 2.2 Experiment 2

#### 2.2.1 Participants

Sixteen undergraduate students (11 females, 5 males; age range: 19–36; mean age: 26 years) from the University of Haifa performed Experiment 2. Two students also participated in Experiment 1. One participant was excluded due to a too-low overall-performance score (0.53). All participants were naïve to the purpose of the study and reported normal or corrected-to-normal vision.

#### 2.2.2 Stimuli apparatus and procedure

Stimuli apparatus and procedure were similar to those of Experiment 1 except for the following: The SOAs were within the temporal crowding range: 175, 225, 275, 375, and 475 ms, and each orientation stimulus was presented for 75 ms, as in Tkacz-Domb and Yeshurun (2021; Experiments 1 and 3). Target and distractors were of different luminance: on half of the trials the target was black (0.01 cd/m2) with white distractors (100 cd/m2) and on the other half the target was white with black distractors. These two types of trials were randomly mixed.

### 2.3 Experiment 3

#### 2.3.1 Participants

Seventeen undergraduate students (13 females, 4 males; age range: 18–35; mean age: 22.3 years) from the University of Haifa performed Experiment 3; one participant also performed Experiment 2 and another two participants performed all three experiments. All participants were naïve to the purpose of the study and reported normal or corrected-to-normal vision. One participant was excluded due to a too-low overall-performance score (0.52).

#### 2.3.2 Stimuli apparatus and procedure

The stimuli apparatus and procedure were similar to those of Experiment 2 except for the following: The possible SOAs were: 40, 60, 80, 100, and 120 ms, and the stimuli duration was 30 ms.

## 3 Results

### 3.1 Experiment 1

**Figure 2:**
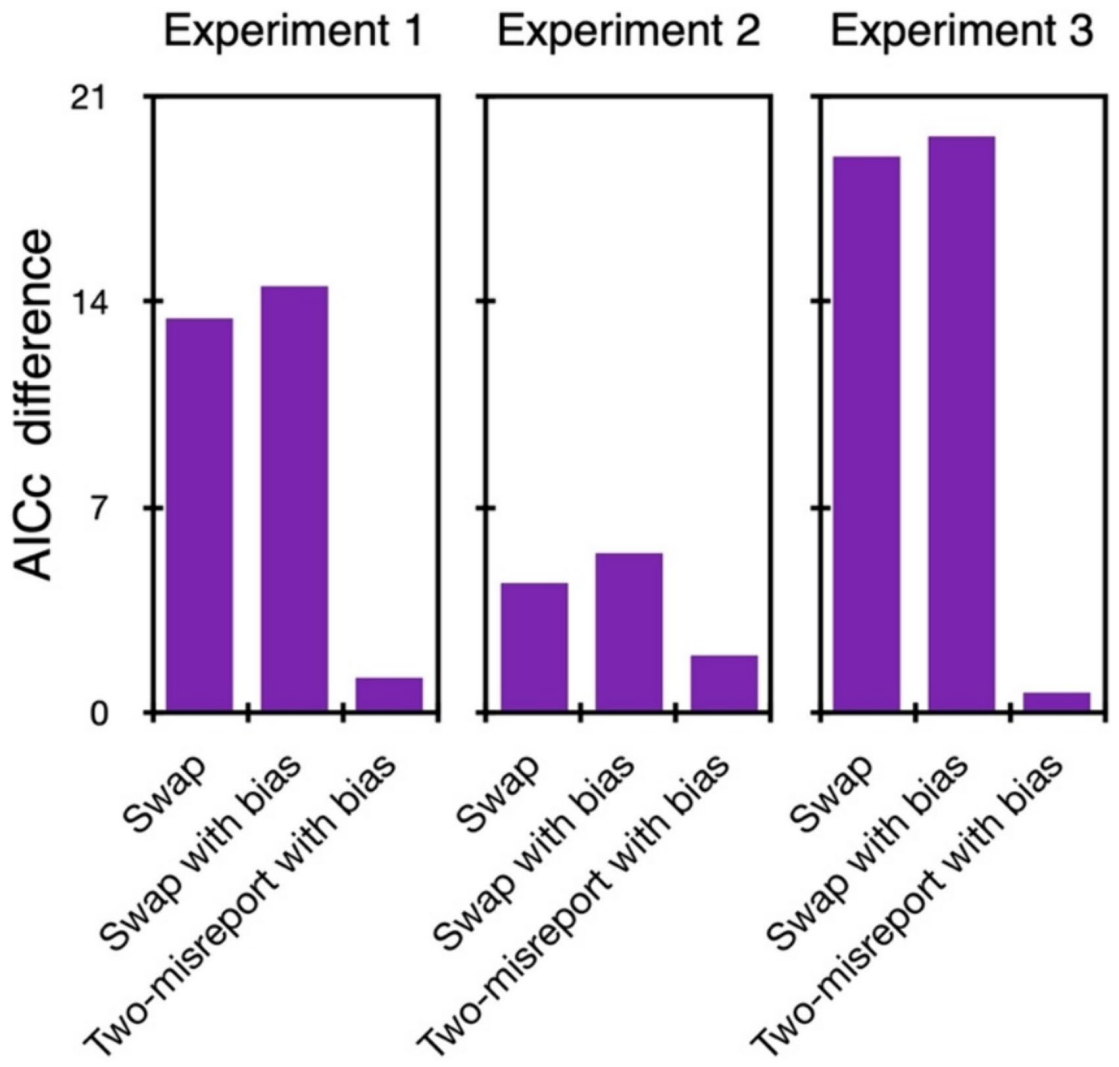
Model comparison in Experiments 1–3. The difference in AICc values between each tested model and the two-misreport mixture model. The two-misreport mixture model had the lowest AICc values (i.e., all differences were positive) in all three experiments.

**Figure 3:**
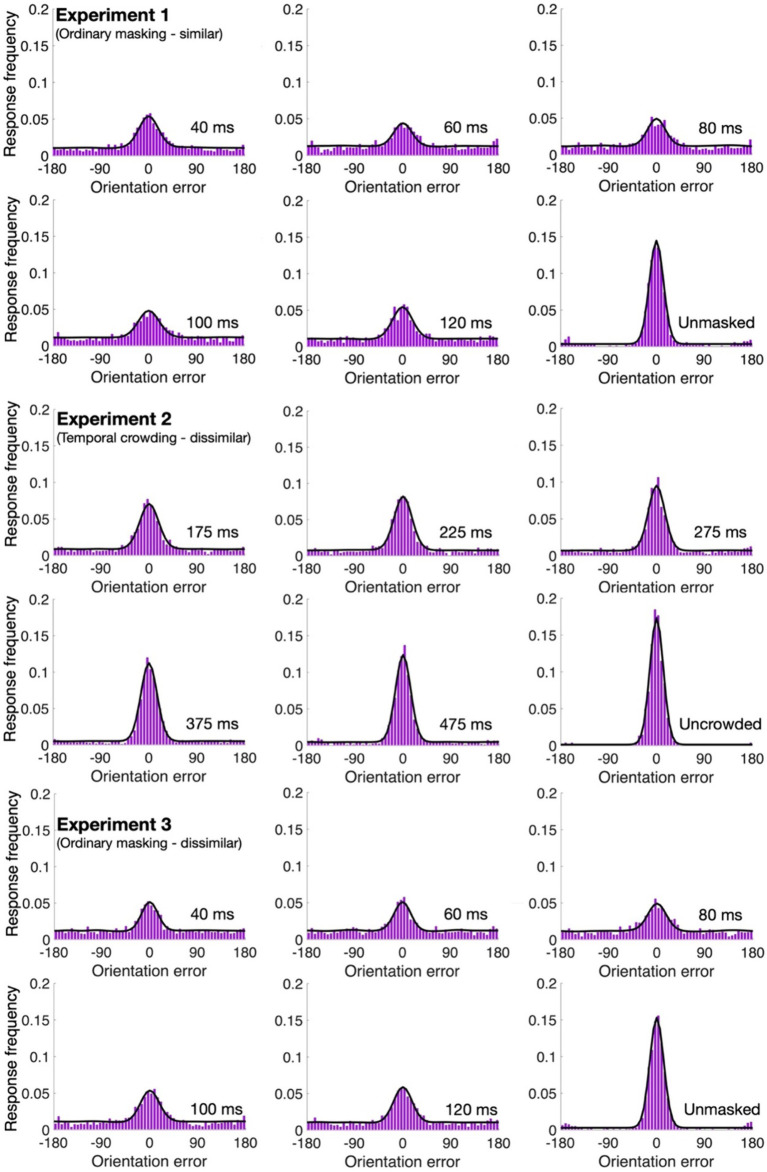
Mean error distributions and model fits (in black) for the various conditions of Experiments 1–3.

4.10% of the trials were excluded from further analyses. As can be seen in Figures 2, 3, with all our experiments, the two-misreport mixture model (Eq. 2) had the lowest AICc values and it fits the data well (examples of the model fit to individual data are provided in the Supplementary material). We therefore proceed with this model.

**Figure 4:**
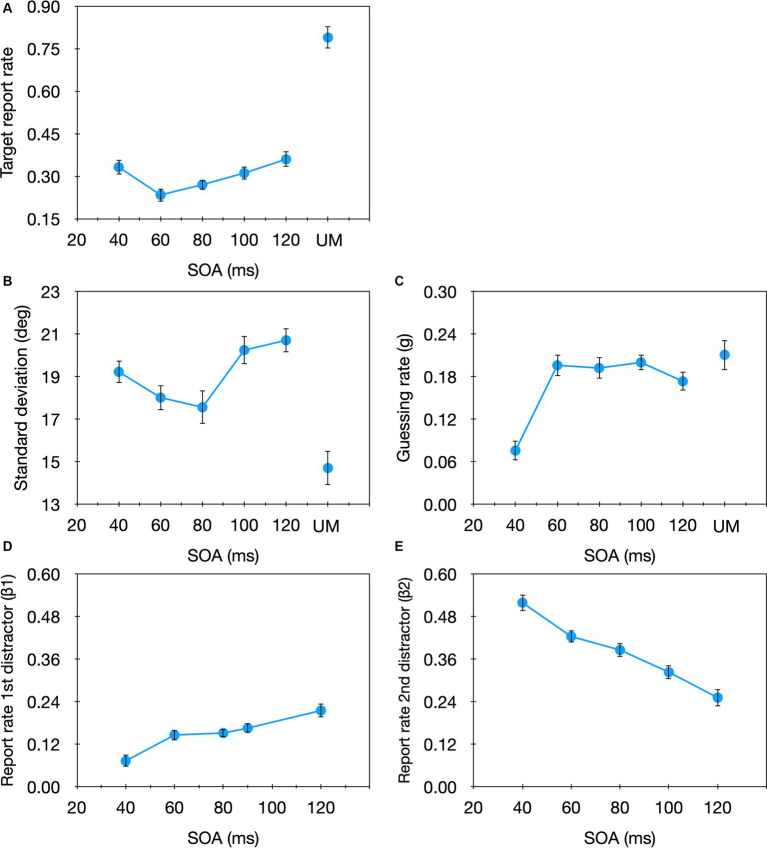
Target report rate **(A)** and the estimated parameters **(B)** *sd*, **(C)** *g*, **(D)** *β_1_*, **(E)** *β_2_* as a function of SOA in the masking condition of Experiment 1. For comparison we also plot the unmasked condition (UM). Error bars represent one standard error.

To evaluate the overall pattern of performance in the current task we start with analyzing the effect of SOA on the proportion of trials in which the participants reported the target’s orientation (i.e., the ‘target report rate,’ reflected in the 1st component of Eq. 2: 1–*g*–*β_1_*–*β_2_*). A one-way (SOA) repeated measures ANOVA revealed a significant SOA effect on the target report rate [*F*(4,60) = 5.22, *p* = 0.001, *η*_p_^2^ = 0.26]. As can be seen in Figure 4A, we obtained the classical non-monotonic (e.g., Alpern, 1953) function typically found when the strength of the target is similar to that of the mask (for reviews and modeling see Francis, 2003; Breitmeyer and Öğmen, 2006). To examine which of the model parameters are affected by masking we performed a one-way (SOA) repeated measures ANOVA on each of the model parameters (Figures 4B–E; individual data are shown in the Supplementary material). No significant SOA effect was found for the *sd* [*F*(4,60) = 2.029, *p* = 0.102, *η*_p_^2^ = 0.119], but a significant effect emerged for the *g* parameter [*F*(4,60) = 5.229, *p* = 0.001, *η*_p_^2^ = 0.259]. This SOA effect seems to be mostly due to the shortest SOA in which the guessing rate was considerably smaller compared with the larger SOAs. This pattern is consistent with the results of Agaoglu et al. (2015) who used a similar estimation task but with only a single mask. Like here, they also found a significant SOA effect for the guessing rate but not the *sd*. Importantly, this pattern is opposite to that found for temporal crowding (Tkacz-Domb and Yeshurun, 2021; Sahar and Yeshurun, 2024), in which significant SOA effects are found for the *sd* but not for the *g* parameter. We also found a significant SOA effect for *β_1_* [*F*(4,60) = 6.242, *p* < 0.001, *η*_p_^2^ = 0.294] and *β_2_* [*F*(4,60) = 23.01, *p* < 0.001, *η*_p_^2^ = 0.605], however, their effects were opposite: With *β_1_*, substitution errors increased with increasing SOA while with *β_2_*, substitution errors decreased with increasing SOA. These results are inconsistent with Agaoglu et al. (2015) who did not find considerable substitution errors with masking. Critically, these results also differ from those obtained for temporal crowding in which the substitution rate decreased as the SOA got longer, for both distractors (Tkacz-Domb and Yeshurun, 2021; Sahar and Yeshurun, 2024).

#### 3.1.1 Direct comparison of masking and temporal crowding

The above analysis shows that the pattern of SOA effects observed for the 4 parameters is very different for masking and temporal crowding, suggesting that these are two different phenomena. In this section, we further test this conclusion by performing an analysis that combines both. Specifically, we compare the data obtained in this experiment with the data obtained in the second experiment of it Tkacz-Domb and Yeshurun (2021), which used identical stimuli but measured temporal crowding and therefore used longer SOAs (170–470 ms). Additionally, stimuli presentation was slightly shorter— 20 ms instead of the current 30 ms, but that study has shown that the pattern of temporal crowding, with all four parameters, remains the same with much larger variations in stimuli duration (75 ms vs. 20 ms). Other than that, the masking and temporal crowding experiments are identical. Because the two experiments use non-overlapping ranges of SOA, we could not analyze the combined data using ANOVA. Instead, we fitted a piecewise regression model to the combined data of each parameter in order to test whether the SOA effect (i.e., the slope) found for a given parameter differs significantly for masking (SOA range: 40–120 ms) and temporal crowding (SOA range: 170–470 ms).

**Figure 5:**
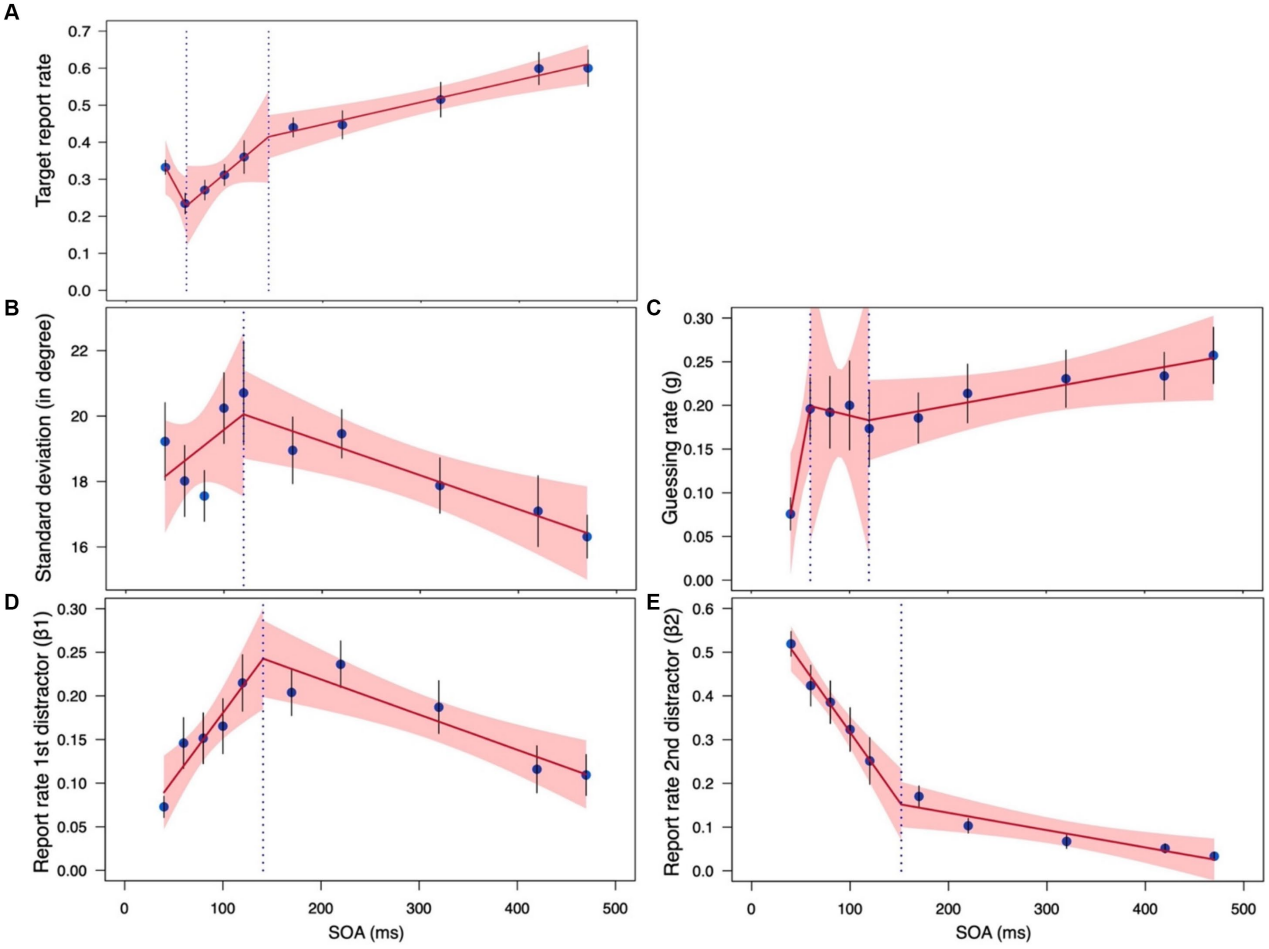
A piecewise regression model fitted to the estimated parameters in the masked/crowded conditions of current Experiment 1 (SOA range: 40–120 ms) and Experiment 2 of Tkacz-Domb and Yeshurun (2021; SOA range:170–470): **(A)** *Target report rate;* **(B)** *sd*; **(C)** *g*; **(D)** *β_1_*; **(E)** *β_2_*. The dotted line shows the breakpoint. Error bars represent one standard error. The shaded region corresponds to 95% CIs.

For the *sd*, *β_1_,* and *β_2_* parameters we could fit a piecewise regression model with a single breakpoint (Figures 5B,D,E). The breakpoints obtained by the regression model are 120, 141, and 152 ms for *sd*, *β_1_*, and *β_2_*, respectively, confirming that the limit of masking is around 100–150 ms (e.g., Breitmeyer and Öğmen, 2000, 2006; Enns and Di Lollo, 2000; Enns, 2004). Additionally, the regression analyses for these parameters confirm the results of the separate ANOVAs: (a) The SOA slope with *sd* was not significant for the masking range (slope = 0.024, *SE* = 0.023, *t* = 1.019 CI_95%_ = −0.022, 0.070) but was significant for the temporal crowding range (slope = −0.010, *SE* =0.003, *t* = −3.189 CI_95%_ = −0.017, −0.004); (b) The SOA slope with *β_1_* was significant for both the masking range (slope = 0.002, *SE* = 0.0004, *t* = 3.470 CI_95%_ = 0.001, 0.002) and the temporal crowding range (slope = −0.0004, *SE* = 0.0001, *t* = −3.833 CI_95%_ = −0.001, −0.0002), but of opposite directions; and (c) The SOA slope with *β_2_* was also significant for both the masking range (slope = −0.003, *SE* = 0.001, *t* = −5.930 CI_95%_ = −0.004, −0.002) and the temporal crowding range (slope = −0.0004, *SE* = 0.0001, *t* = −3.064 CI_95%_ = −0.001, −0.0001), though it was tenfold steeper in the masking case. Critically, with all 3 parameters, the SOA slope of the masking range was significantly different from the SOA slope of the temporal crowding range, supporting the conclusion that these are different phenomena that are mediated by different processes [*sd*: *F*(1,161) = 4.709, *p* = 0.032; *β_1_*: *F*(1,161) = 27.634, *p* < 0.001; *β_2_*: *F*(1,161) = 45.362, *p* < 0.001]. We further note that with all 3 parameters, the overall model fit of the piecewise regression analysis was statistically significant [*sd*: *F*(3,161) = 3.97, *p* = 0.009; *β_1_*: *F*(3,161) = 9.377, *p* < 0.001; *β_2_*: *F*(3,161) = 80.019, *p* < 0.001].

**Figure 6:**
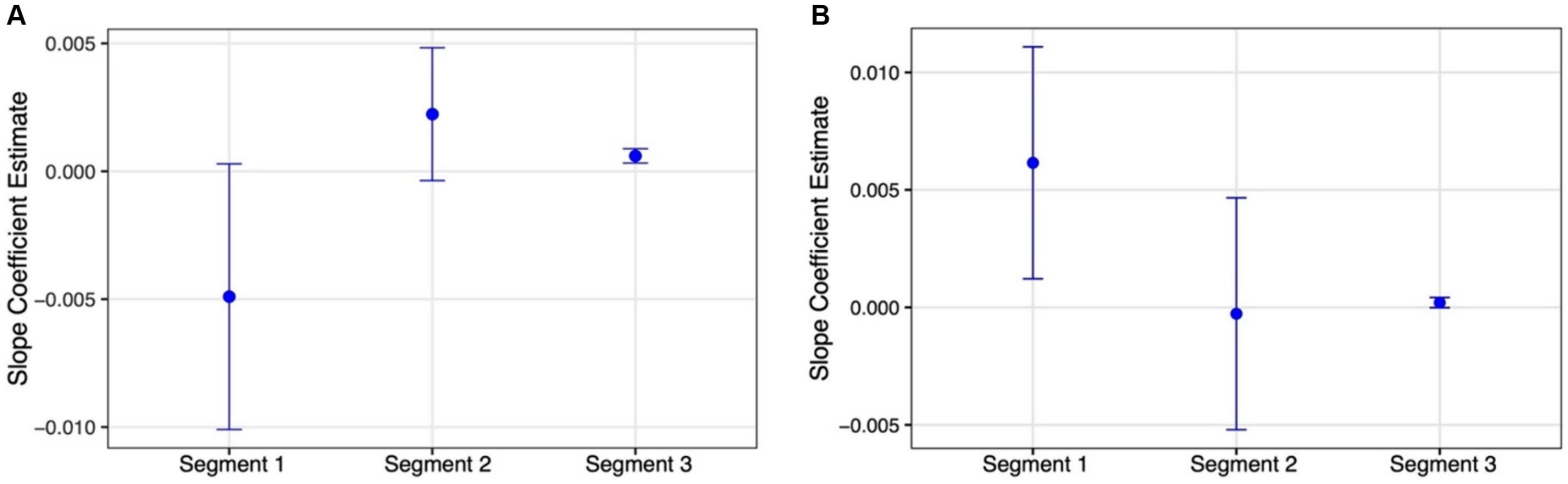
Estimated slopes and 95% CIs (error bars) for the 3 SOA segments generated by the piecewise regression model for the: **(A)** *Target report rate;* **(B)** *g* parameter (see text).

With the target report rate and the *g* parameter, however, we could not fit a model with a single breakpoint. This is likely because of the non-monotonic nature of their function in the masking part. Thus, instead, we fit their data with a model that allows for two breakpoints (Figures 5A,C). The fit of this model was statistically significant [Target report rate: *F*(5,159) = 22.809, *p* < 0.0001; *g*: *F*(5,159) = 3.464, *p* = 0.005] and, as expected, it revealed one breakpoint that marks the difference in target report rater and *g* between the shortest SOA and the other SOAs within the masking range (61 and 60 ms, respectively), and another breakpoint marking the difference between the masking and temporal crowding SOA ranges (145 and 120 ms, respectively). A slope analysis for the target report rate indicated that the slope of the third segment was significant (slope3 = 0.0006, *SE* = 0.0001, *t* = 4.259 CI_95%_ = 0.0003, 0.0009), while those of the other segments were not (slope1 = −0.0049, *SE* = 0.0026, *t* = −1.863 CI_95%_ = −0.0101, 0.0003; slope2 = 0.0022, *SE* = 0.0013, *t* = 1.699 CI_95%_ = −0.0004, 0.0048). The opposite pattern emerged for the *g* parameter. The slope of the first segment was significant (slope1 = 0.006, *SE* = 0.003, *t* = 2.461 CI_95%_ = 0.001, 0.011), while those of the other segments were not (slope2 = −0.0003, *SE* = 0.003, *t* = −0.109 CI_95%_ = −0.005, 0.005; slope3 = 0.0002, *SE* = 0.0001, *t* = 1.842 CI_95%_ = −0.00002, 0.0004). Importantly, as is apparent by the non-overlapping CIs in Figure 6, the slope for the temporal crowding segment (segment 3) is reliably different from that of the first masking segment (segment 1).

### 3.2 Experiment 2

**Figure 7:**
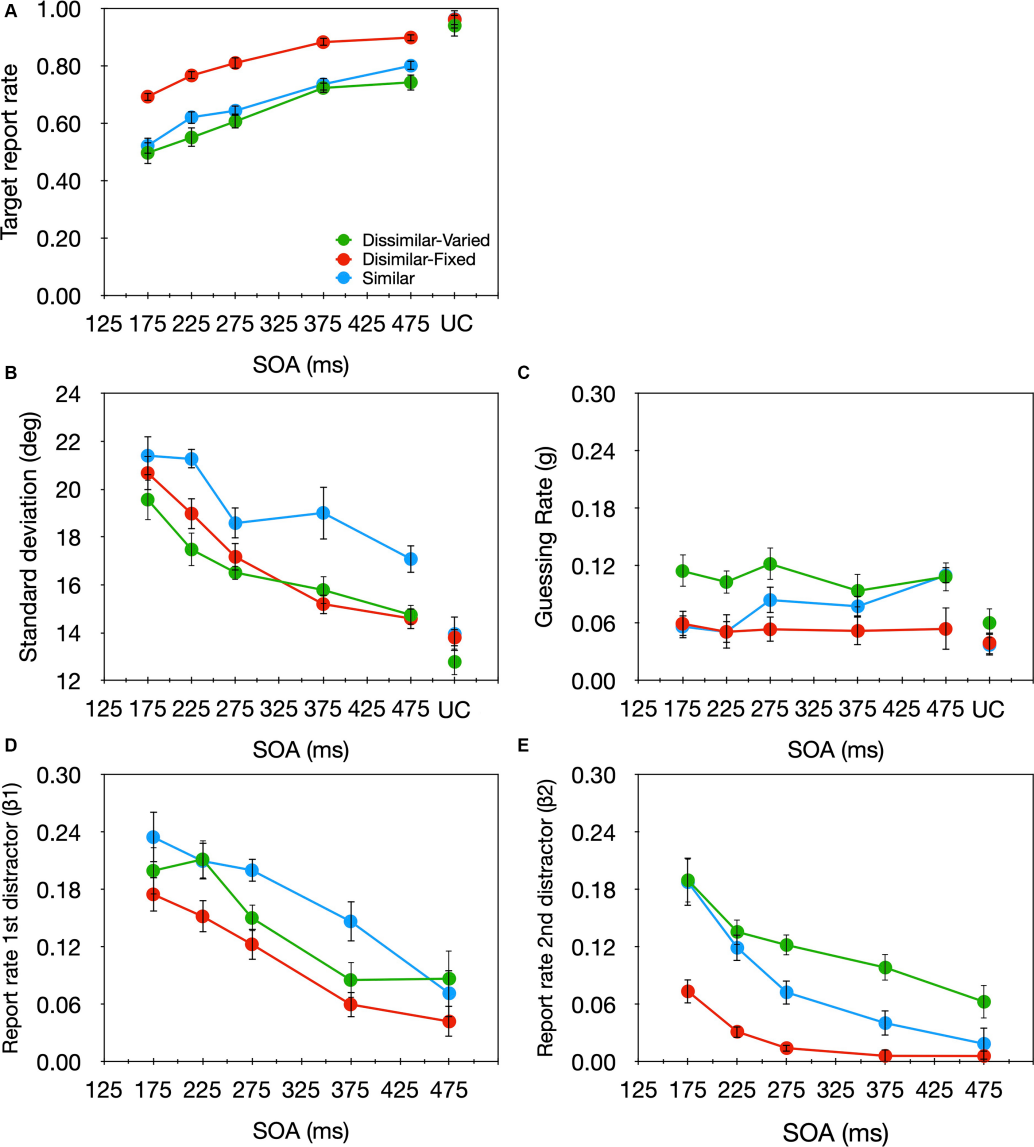
Target report rate **(A)** and the estimated parameters **(B)** sd, **(C)** g, **(D)** β_1_, **(E)** β_2_ as a function of SOA in the crowded condition, and the uncrowded condition (UC) in: Experiment 2 of the current study where stimulus luminance varied randomly (green), Experiment 1 of Tkacz-Domb and Yeshurun (2021) where all stimuli had similar luminance (blue), and Experiment 3 of Tkacz-Domb and Yeshurun (2021) where stimulus luminance was fixed throughout the experiment (red).

2.95% of the trials were excluded from further analyses. A one-way (SOA) repeated-measures ANOVA revealed that the target report rate increased significantly as the SOA increased [*F*(4,56) = 14.08, *p* < 0.001, *η*_p_^2^ = 0.501; Figure 7A green curve]. A similar analysis on each of the model parameters (Figures 7B–E, green curves) revealed a significant effect of SOA for the *sd*, *β_1_*, and *β_2_* parameters [*F*(4,56) = 9.51, *p* < 0.001, η_p_^2^ = 0.405; *F*(4,56) = 7.605, *p* < 0.001, *η*_p_^2^ = 0.352; *F*(4,56) = 8.487, *p* < 0.001, *η*_p_^2^ = 0.377, respectively], but not the *g* parameter [*F*(4,56) = 0.461, *p* = 0.764, *η*_p_^2^ = 0.032]. With relatively short SOAs (i.e., strong temporal crowding) *sd*, *β_1_*, and *β_2_* were high, suggesting that the precision of the target’s encoding was low and the substitution rate with both preceding and succeeding distractors was high. As the SOA got longer (i.e., temporal crowding decreased) precision was enhanced and substitution errors were reduced. This is in line with the pattern of effects found thus far for temporal crowding (Tkacz-Domb and Yeshurun, 2021; Sahar and Yeshurun, 2024). Also consistent with previous findings, even with an SOA of 475 ms, the *sd* was significantly larger than that observed in the uncrowded condition [*t*(14) = 2.798, *p* = 0.014, Cohens *d* = 0.76], demonstrating once again that the impairment caused by temporal crowding lasts for a particularly long time.

#### 3.2.1 Temporal crowding with similar vs. dissimilar target and distractors

To examine directly the effect of target–distractor similarity on temporal crowding we compared the effects of SOA on each parameter when the target and distractors had different luminance—current experiment, and when they had the same luminance—Experiment 1 from Tkacz-Domb and Yeshurun (2021; Figure 7, green and blue curves, respectively). All other stimuli parameters were identical in both experiments. We conducted a two-way mixed-design ANOVA on each parameter, with SOA as a within-subject variable and similarity (similar vs. dissimilar) as a between-subject variable. As expected given previous analyses, a significant main effect of SOA emerged for the *sd*, *β_1_,* and *β_2_* parameters [*F*(4,112) = 14.022, *p* < 0.001, *η*_p_^2^ = 0.334; *F*(4,112) = 15.766, *p* < 0.001, *η*_p_^2^ = 0.360; *F*(4,112) = 23.532, *p* < 0.001, *η*_p_^2^ = 0.457, respectively], but not for the g parameter [*F*(4,112) = 1.364, *p* = 0.251, *η*_p_^2^ = 0.046]. Critically, there was a significant similarity effect for the *sd* [*F*(1,28) = 4.751, *p* = 0.038, *η*_p_^2^ = 0.145]; the *sd* was smaller—encoding precision was higher—when the target and distractors differed in luminance. The target-distractor similarity did not affect the other parameters [*β_1_: F*(1,28) = 0.689, *p* = 0.414, *η*_p_^2^ = 0.024, *β_2_: F*(1,28) = 0.573, *p* = 0.455, *η*_p_^2^ = 0.020, *g: F*(1,28) = 1.143, *p* = 0.294, *η*_p_^2^ = 0.039], and there was no significant SOA × Similarity interaction for either of the parameters [*sd*: *F*(4,112) = 0.739, *p* = 0.567, *η*_p_^2^ = 0.026; *β_1_: F*(4,112) = 1.186, *p* = 0.321, *η*_p_^2^ = 0.041; *β_2_: F*(4,112) = 1.016, *p* = 0.402, *η*_p_^2^ = 0.035; *g: F*(4,112) = 1.162, *p* = 0.331, *η*_p_^2^ = 0.040]. These effects of similarity are very different from those found with fixed target-distractor luminance values (Figure 7, red curve). With fixed luminance values, there was a significant effect of similarity for *β_1_* and *β_2_* but not for *sd*. These differences in similarity effects will be discussed in the General Discussion section.

### 3.3 Experiment 3

**Figure 8:**
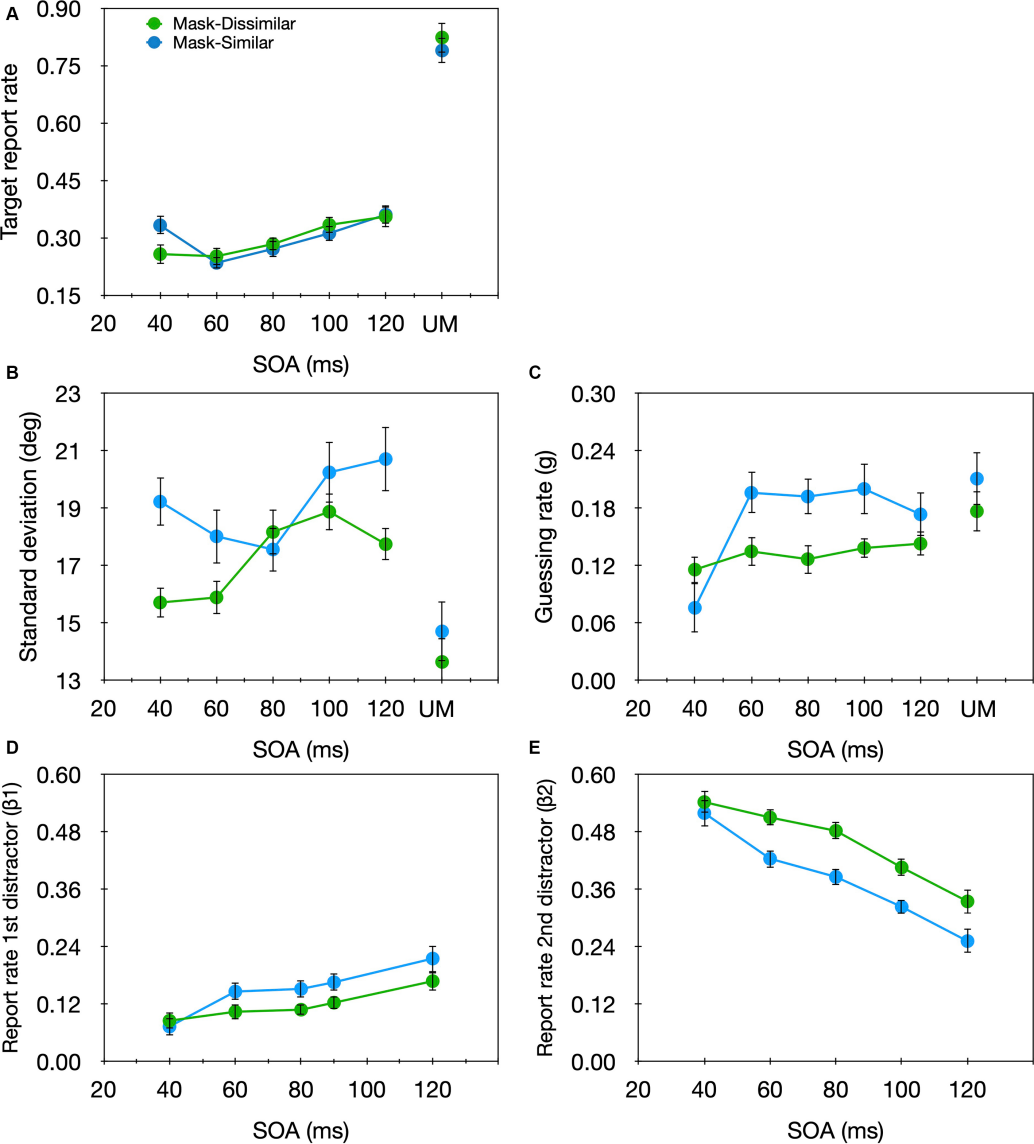
Target report rate **(A)** and the estimated parameters **(B)** *sd*, **(C)** *g*, **(D)** *β_1_*, **(E)** *β_2_* as a function of SOA in the masked condition, and the unmasked condition (UM) in Experiment 1 (Mask-Similar; blue), and Experiment 3 (Mask-Dissimilar; green).

The results were analyzed similarly to Experiment 1 (Figure 8, green curves). 3.9% of the trials were excluded from further analyses. A significant SOA effect was found for the target report rate [*F*(4,60) = 5.10, *p* = 0.001, *η*_p_^2^ = 0.26]. Unlike the non-monotonic pattern found when the target and masks shared the same luminance, here, the target report rate increased monotonically with the SOA. Also, we found a significant SOA effect for the *sd* [*F*(4,60) = 5.231, *p* = 0.001, *η*_p_^2^ = 0.259] but not for the *g* parameter [*F*(4,60) = 0.67, *p* = 0.615, *η*_p_^2^ = 0.043]. This pattern is practically opposite to that found in Experiment 1 and might seem similar to that of temporal crowding, however, with temporal crowding the *sd* decreased with SOA whereas here it increased with the SOA, suggesting that target encoding precision was reduced rather than improved with larger SOAs. As for substitution errors, the results were similar to Experiment 1. A significant main effect of SOA emerged for both *β_1_* [*F*(4,60) = 4.161, *p* = 0.005, *η*_p_^2^ = 0.217] and *β_2_* [*F*(4,60) = 16.795, *p* < 0.001, *η*_p_^2^ = 0.528], but of opposite directions: *β_1_* increased with SOA while *β_2_* decreased with SOA.

#### 3.3.1 Masking with similar vs. dissimilar target and distractors

We conducted a two-way mixed design ANOVA on each of the parameters generated for Experiments 1 and 3, with SOA as a within-subject variable and similarity (similar vs. dissimilar) as a between-subject variable. A significant main effect of SOA emerged for the *sd* [*F*(4,120) = 3.935, *p* = 0.005, *η*_p_^2^ = 0.116], *g* [*F*(4,120) = 5.340, *p* < 0.001, *η*_p_^2^ = 0.151], *β_1_* [*F*(4,120) = 10.019, *p* < 0.001, *η*_p_^2^ = 0.250], and *β_2_* [*F*(4,120) = 39.000, *p* < 0.001, *η*_p_^2^ = 0.565] parameters. There was no significant main effect of similarity for either of the parameters [*sd*: *F*(1,30) = 3.246, *p* = 0.082, *η*_p_^2^ = 0.098]; *g*: [*F*(1,30) = 0.652, *p* = 0.426, *η*_p_^2^ = 0.021]; *β_1_*: [*F*(1,30) = 1.162, *p* = 0.290, *η*_p_^2^ = 0.037]; *β_2_*: [*F*(1,30) = 1.732, *p* = 0.198, *η*_p_^2^ = 0.055]. However, a significant SOA × similarity interaction was found for the *g* parameter [*F*(4,120) = 2.874, *p* < 0.026, *η*_p_^2^ = 0.087]; the effect of SOA was significant only when the target and distractors had similar luminance. No other interaction was significant [*sd*: *F*(4,120) = 1.997, *p* = 0.099, *η*_p_^2^ = 0.062]; *β_1_*: [*F*(4,120) = 0.976, *p* = 0.423, *η*_p_^2^ = 0.032]; *β_2_*: [*F*(4,120) = 0.980, *p* = 0.421, *η*_p_^2^ = 0.032]. Thus, unlike temporal crowding in which similarity mainly affects the encoding precision—the encoding precision is lower when the target and the distractors are similar, with masking, similarity mainly affects the SNR—the SNR increases with SOA only when the target and the distractors are similar.

## 4 General discussion

The current study examined interference across time, and specifically, how it varies as a function of its temporal scale. To that end, we performed a direct comparison between temporal interference over short intervals—masking—and temporal interference over longer intervals—temporal crowding. Additionally, we compared how the interference brought about by the two phenomena is modified by target-distractor similarity. Importantly, to better understand which perceptual aspects are impaired by each phenomenon, in all experiments, the error distributions of an orientation estimation task were analyzed using the two-misreport mixture model (Shechter and Yashar, 2021).

The results of the three experiments clearly demonstrate that temporal crowding and masking generate different patterns of effects on encoding precision, guessing rate, and substitution errors. This suggests that temporal interference of different scales is mediated by different mechanisms. Masking affects the SNR but not the encoding precision. In contrast, temporal crowding mainly affected the encoding precision, but not the SNR. Likely, when the inter-item intervals are short, as is the case with masking, the interference is mainly mediated by processes occurring at early processing stages like faulty integration of the target’s and distractors’ signals or direct inhibition of the target’s signal by that of the distractors, which reduces the SNR. Indeed, Agaoglu et al. (2015) conclude, based on the non-monotonic masking function, that the reduced SNR they observed for pattern masking by structure reflects both increased noise and reduced signal. Such ‘early’ target-distractor integration/inhibition is less likely with temporal crowding, given the relatively long inter-item intervals, and accordingly there is no evidence for modified SNR.

Different patterns of results also emerged for substitution errors. With temporal crowding, substitution errors decreased as the SOA increased for both distractors. This is probably because as the SOA increases uncertainty regarding the order of the items in the sequence decreases thereby reducing the probability of mistakenly reporting a distractor. Substitution errors also decreased with SOA for the second distractor of masking, however, the slope of this decrease was tenfold steeper than temporal crowding, further supporting the conclusion that they reflect different processes. In contrast, with the first distractor of masking, substitution errors increased with SOA. Perhaps, with very short SOAs, the first distractor was hardly registered in sensory memory because the two following items elicited early integration/inhibition processes. This prevented confusing it with the target, and consequently, substitution errors were primarily determined by the second distractor. With longer SOAs, the encoding of the first distractor into sensory memory was improved, thereby increasing the probability of confusing it with the target, while decreasing the probability of confusing the second distractor with the target. In contrast, with temporal crowding, all three items were registered preventing such trade-offs.

Similarity effects also differed for the two phenomena. With masking, similarity mainly affected the SNR; the effect of SOA on the guessing rate was only significant when the target and distractors shared the same luminance. This is consistent with the possibility that masking effects on the SNR are related to early integration/inhibition processes because when the target and the distractors have different luminance with opposite contrast polarity (i.e., white vs. black on a gray background) they are likely processed by different processing channels that interact to a lesser degree. Importantly, the effects of similarity on temporal crowding were considerably different from those observed for masking. With temporal crowding, similarity mainly affected the encoding precision, with no effect on the SNR: the *sd* was larger with similar target and distractors. Here too, similar distractors may be encoded by the same processing channels as the target thereby reducing the precision of its encoding. Interestingly, unlike Tkacz-Domb and Yeshurun (2021), we did not find similarity effects on the substitution rate in temporal crowding. In that study, the target was marked by a unique feature allowing the participants to adopt higher-level strategies (e.g., report the black item) to reduce source confusion. Such high-level strategies could not be utilized in the current study because the luminance varied randomly. Thus, the lack of this dissimilarity benefit in our study implies that this benefit indeed relied on such higher-level strategies. Still, even with dissimilar distractors, temporal crowding reliably impaired the encoding precision. Thus, when considering current and previous findings (Tkacz-Domb and Yeshurun, 2021; Sahar and Yeshurun, 2024), it becomes clear that the degradation of target representation (i.e., reduced precision) is the most prominent and robust characteristic of temporal crowding; it is found with different stimuli durations, both at the fovea and the periphery, regardless of target-distractor similarity, and even when the distractors do not include orientation information. Moreover, even when the target and distractors were dissimilar, we still found degraded encoding precision with the longest SOA (475 ms). This finding adds to previous findings showing such long-lasting impairment brought about by temporal crowding (Tkacz-Domb and Yeshurun, 2021; Sahar and Yeshurun, 2024), and it is consistent with other demonstrations of long-lasting temporal interactions (Otto et al., 2009; Scharnowski et al., 2009). Together, these findings qualify the common notion that the visual system can represent visual information within ~150 ms (e.g., Thorpe et al., 1996; Bacon-Mace et al., 2005; Grill-Spector and Kanwisher, 2005; Fei-Fei et al., 2007; Castelhano and Henderson, 2008; Greene and Oliva, 2009). An initial, volatile, visual representation may be generated fast, but our findings suggest that the generation of a robust and stable representation is considerably slower.

Can visual short-term memory decay or capacity limitation account for the pattern of interference found for temporal crowding? Starting with memory decay, if temporal crowding was due to decay in visual short-term memory, we would have expected stronger interference as the SOA increases due to the longer decay. The typical pattern found for temporal crowding (e.g., Tkacz-Domb and Yeshurun, 2021; Sahar and Yeshurun, 2024), however, suggests otherwise: performance improves as the SOA increases. It is also unlikely that memory capacity limitation mediates temporal crowding. The participants were required to remember a single item—the target, and they knew in advance that the target would always be the second item of the sequence. Thus, there was no need to encode the entire sequence. Even if the participants did encode all items, three lines are within the storage capacity of visual short-term memory (e.g., Pashler, 1988; Luck and Vogel, 1997; Wheeler and Treisman, 2002). Critically, the conclusion that memory capacity limitation does not play a central role is further supported by the finding that temporal crowding persisted even when the distractors consisted of a circle with no inner line, such that the only oriented item presented was the target (Sahar and Yeshurun, 2024, Experiment 4). Thus, as discussed above, the SOA effect on precision suggests that temporal crowding reflects interference with the encoding of the stimuli into visual short-term memory rather than memory capacity or maintenance duration limitations.

Finally, it should be stressed that temporal crowding is fundamentally different from the attentional blink phenomenon. A typical attentional blink paradigm consists of a fast (SOAs around 100 ms) serial presentation of stimuli, of which two items are the to-be-identified targets. The blink refers to the poor identification of the *second* target and it is typically attributed to the need to consolidate the representation of the first target into working-memory (reviewed in Snir and Yeshurun, 2017). In contrast, with temporal crowding, the SOAs are considerably longer, and participants are required to report only a *single* target. Therefore, the observed impairment cannot be attributed to the ongoing consolidation of a prior target.

To summarize, we found almost opposite patterns of effects for temporal interference of different scales. Whereas temporal crowding affects the precision of target encoding but not the SNR, masking affects the SNR but not the encoding precision. Both affect substitution errors, but in a different manner. Furthermore, target-distractor similarity decreased encoding precision with temporal crowding but only affected the SNR with masking. Altogether, our results suggest that different mechanisms mediate temporal interference of different scales. These findings have important theoretical and practical implications for theories of human visual perception. They suggest that theories of temporal processing need to incorporate different mechanisms, which operate at different stages of processing, for interference of different scales. Additionally, theories assuming a fast generation of visual representation need to allow representation modification even with intervals approaching half a second. Furthermore, our findings suggest that markedly long inter-item intervals are required to completely avoid interference, though the optimal inter-item interval depends on one’s goals as different aspects of perception are impaired with different intervals. Lastly, utilizing dissimilar items partially alleviates temporal interference, though this should also be combined with optimal inter-item intervals depending on the aspect of perceptual processing one wishes to protect from interference.

## Data availability statement

The datasets presented in this study can be found in online repositories. The names of the repository/repositories and accession number(s) can be found at: https://osf.io/af8xm/?view_only=2590b93e461b455895eaf9ce28759154.

## Ethics statement

The studies involving humans were approved by the ethics committee of the University of Haifa (287/19). The studies were conducted in accordance with the local legislation and institutional requirements. The participants provided their written informed consent to participate in this study.

## Author contributions

IH: Writing – review & editing, Writing – original draft, Methodology, Investigation, Formal analysis, Conceptualization. AA-A: Writing – review & editing, Formal analysis. YY: Writing – review & editing, Methodology, Funding acquisition, Formal analysis, Conceptualization.
