# Supplementary material for: Interference across time: dissociating short from long temporal interference
Source: Front Psychol. 2024 Jul 24;15:1393065. doi: 10.3389/fpsyg.2024.1393065 (PMC11305178; doi:10.3389/fpsyg.2024.1393065)
Supplement: Supplementary file 1 [file Data_Sheet_1.pdf]

Supplementary Material

Interference across time: Dissociating short from long temporal interference

Ilanit Hochmitz*, Ahmad Abu-Akel, Yaffa Yeshurun

*** Correspondence:** Ilanit Hochmitz, ilanit57@gmail.com

Supplementary Figures

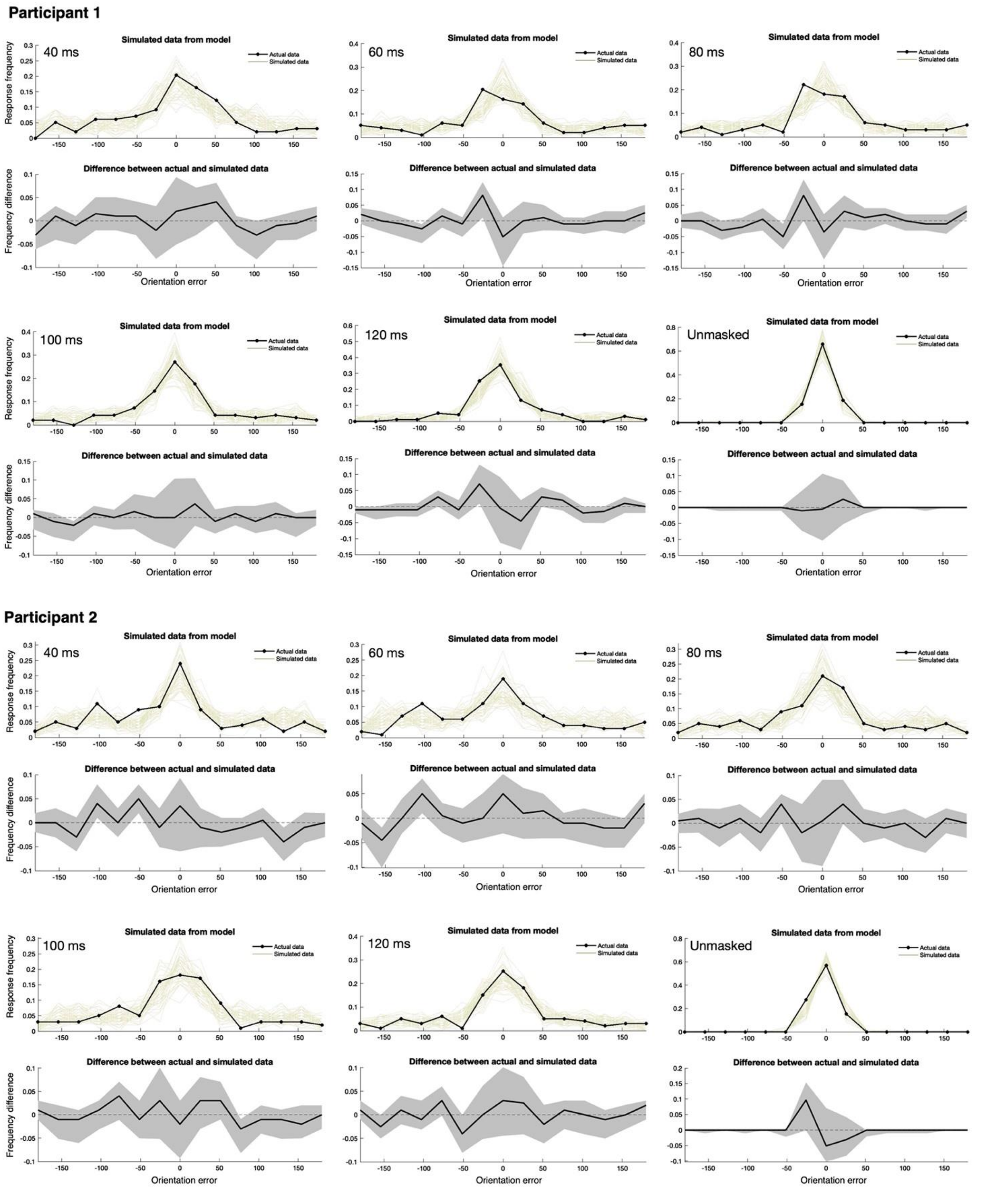

Supplementary Figure 1. Example of diagnostic plots for two randomly chosen participants (P1 and P2) for the various conditions of Experiment 1. The top plot, of each condition, shows the actual data in black and the simulated data in green. The bottom plot shows the average difference between the actual and simulated data (grey line) with 95% confidence intervals (grey shading). Regions in which the gray shading includes zero are regions where the model correctly predicts the data.

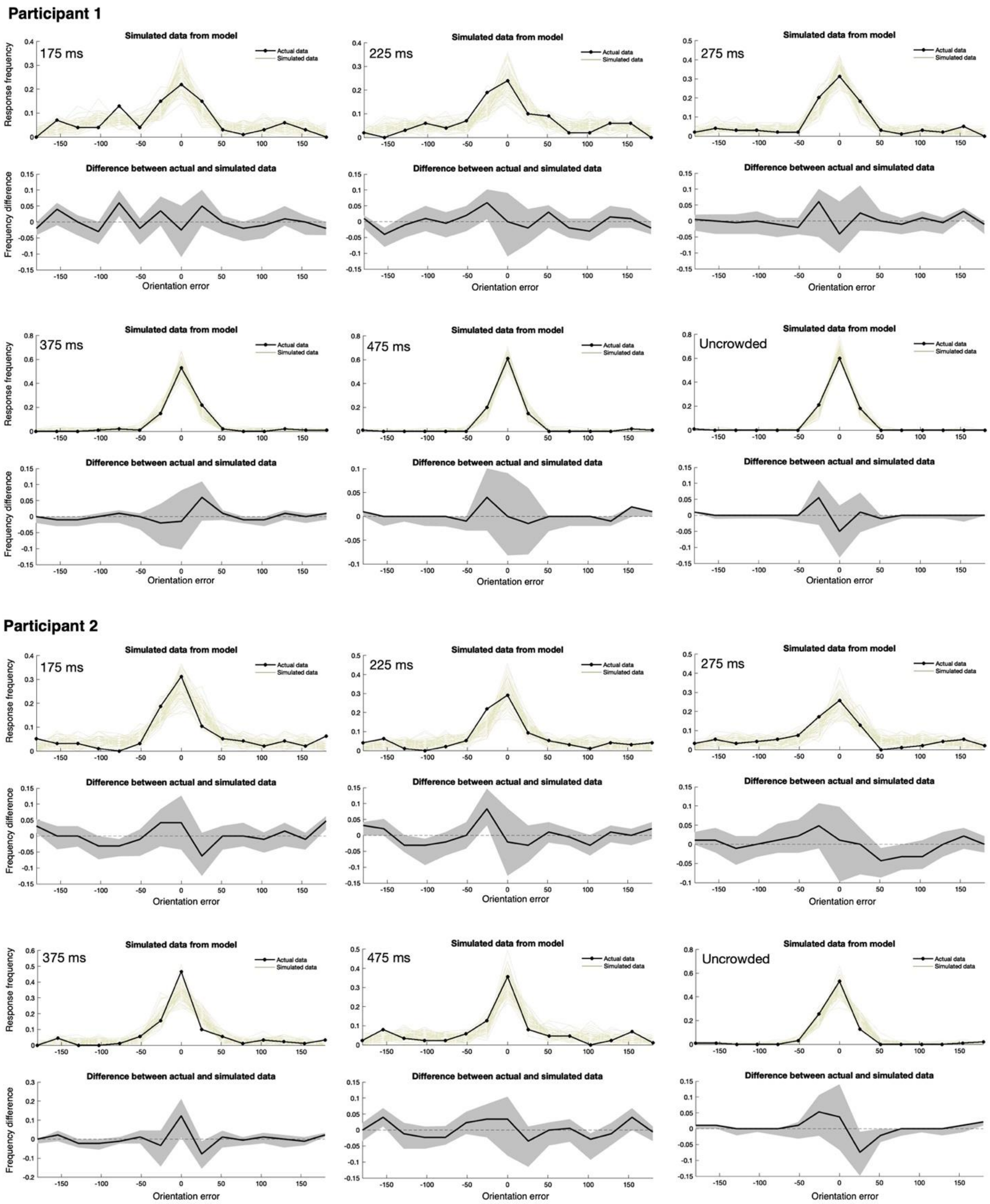

Supplementary Figure 2. Example of diagnostic plots for two randomly chosen participants (P1 and P2) for the various conditions of Experiment 2. The top plot, of each condition, shows the actual data in black and the simulated data in green. The bottom plot shows the average difference between the actual and simulated data (grey line) with 95% confidence intervals (grey shading). Regions in which the gray shading includes zero are regions where the model correctly predicts the data.

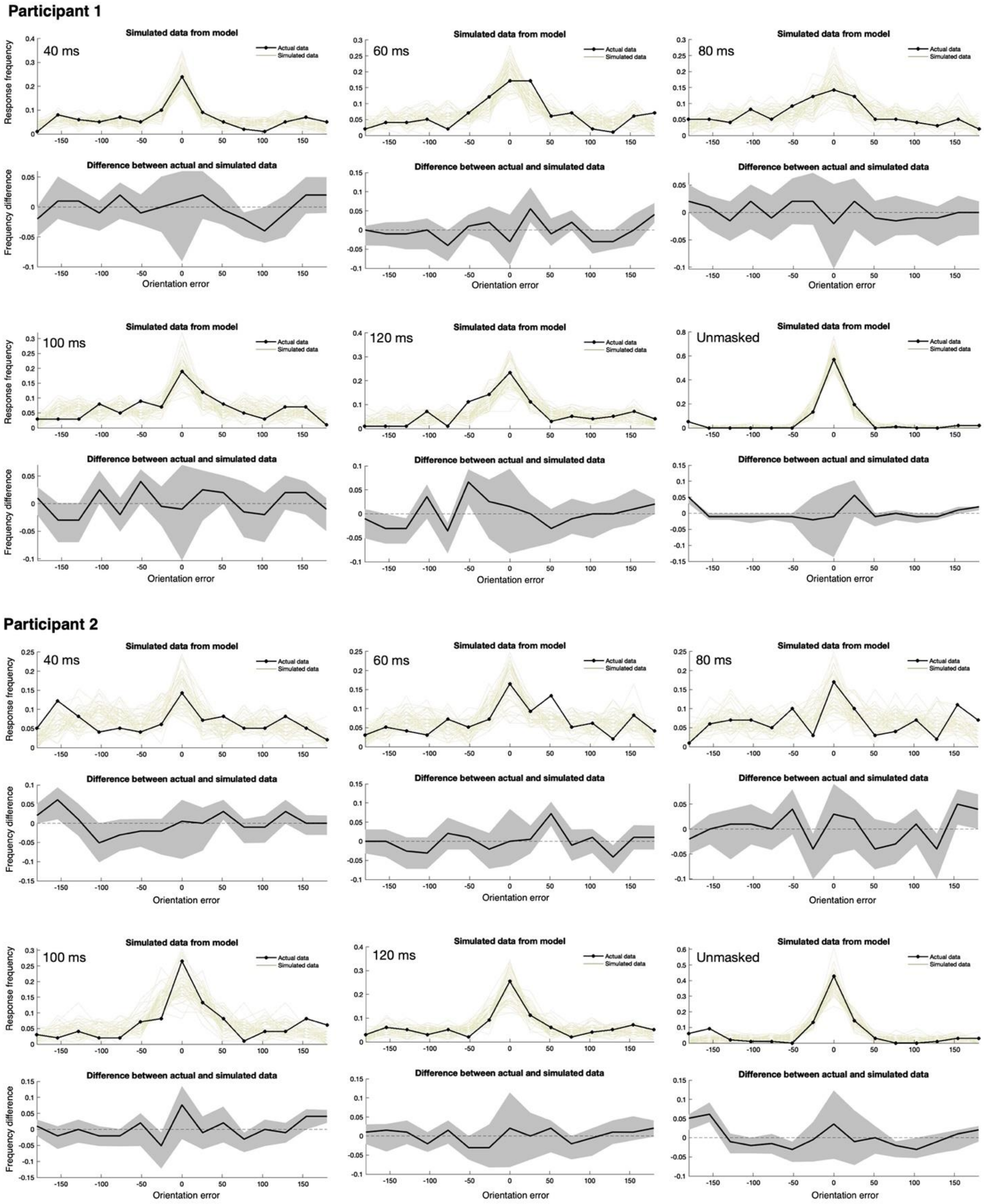

Supplementary Figure 3. Example of diagnostic plots for two randomly chosen participants (P1 and P2) for the various conditions of Experiment 3. The top plot, of each condition, shows the actual data in black and the simulated data in green. The bottom plot shows the average difference between the actual and simulated data (grey line) with 95% confidence intervals (grey shading). Regions in which the gray shading includes zero are regions where the model correctly predicts the data.

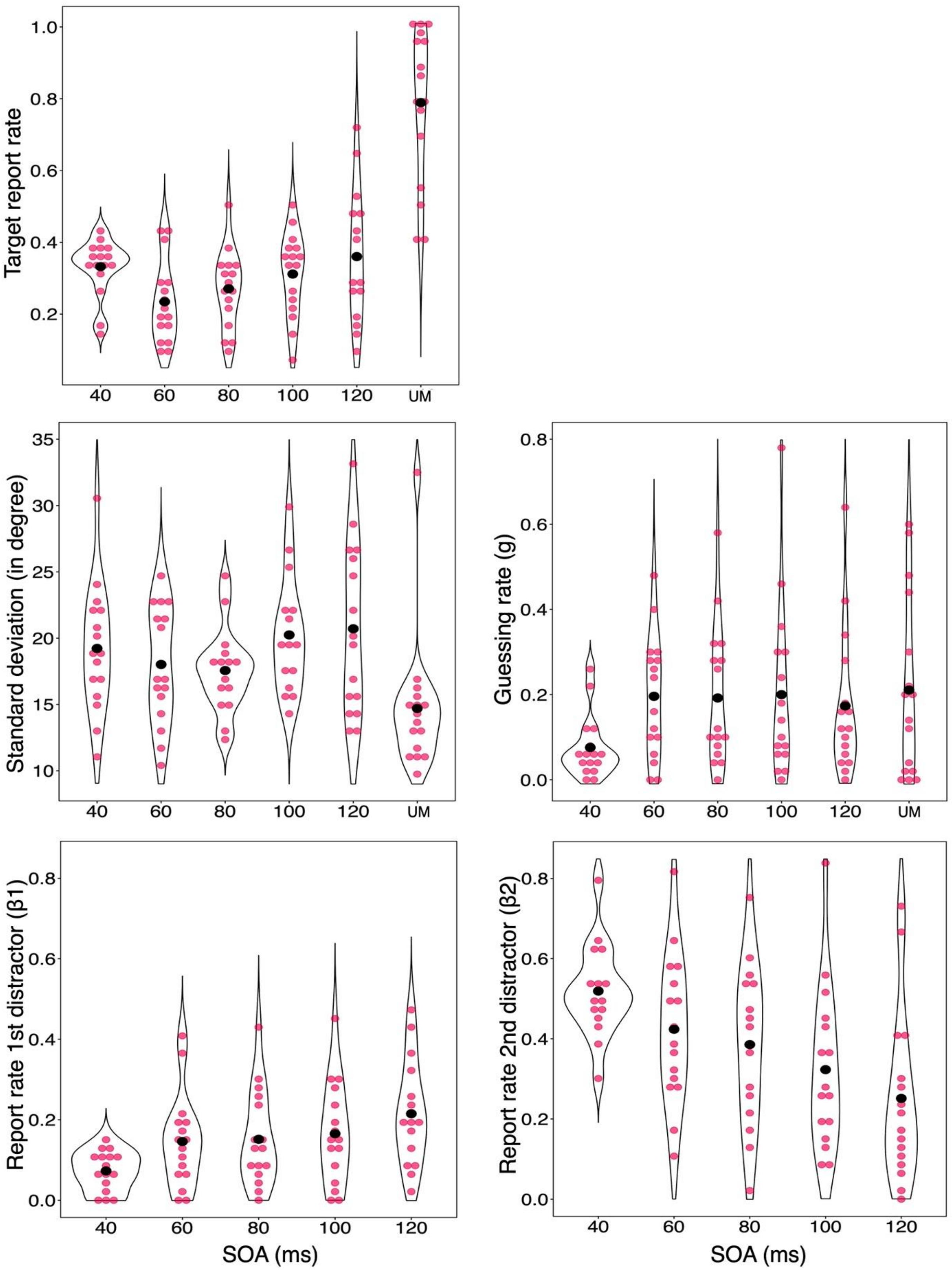

Supplementary Figure 4. Violin plots and dot plots (binned individual data in pink, average in black) of the mixture model parameters as a function of SOA in the masking condition and the unmasked (UM) condition of Experiment 1.

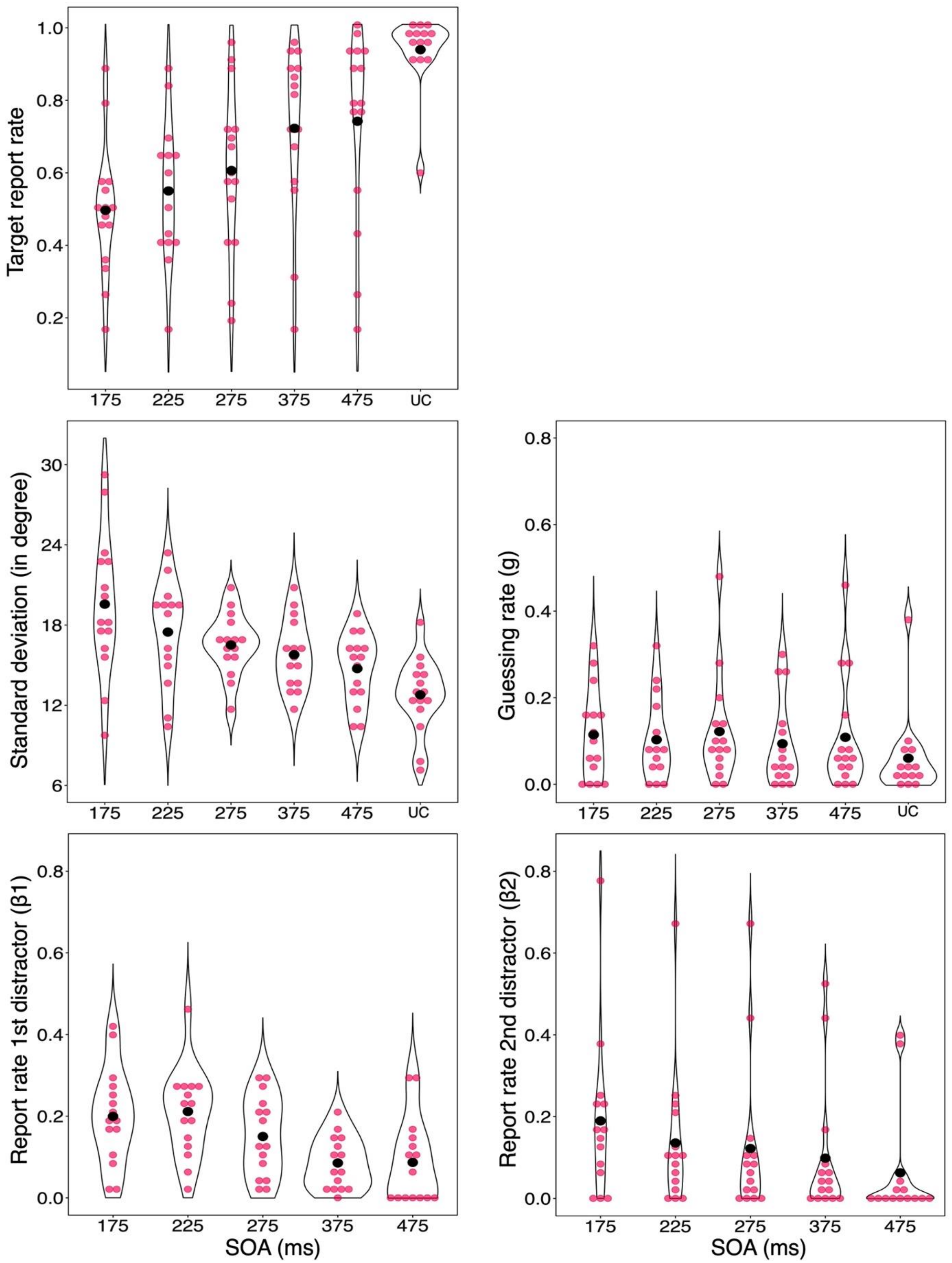

Supplementary Figure 5. Violin plots and dot plots (binned individual data in pink, average in black) of the mixture model parameters as a function of SOA in the crowded condition and the uncrowded (UC) condition of Experiment 2.

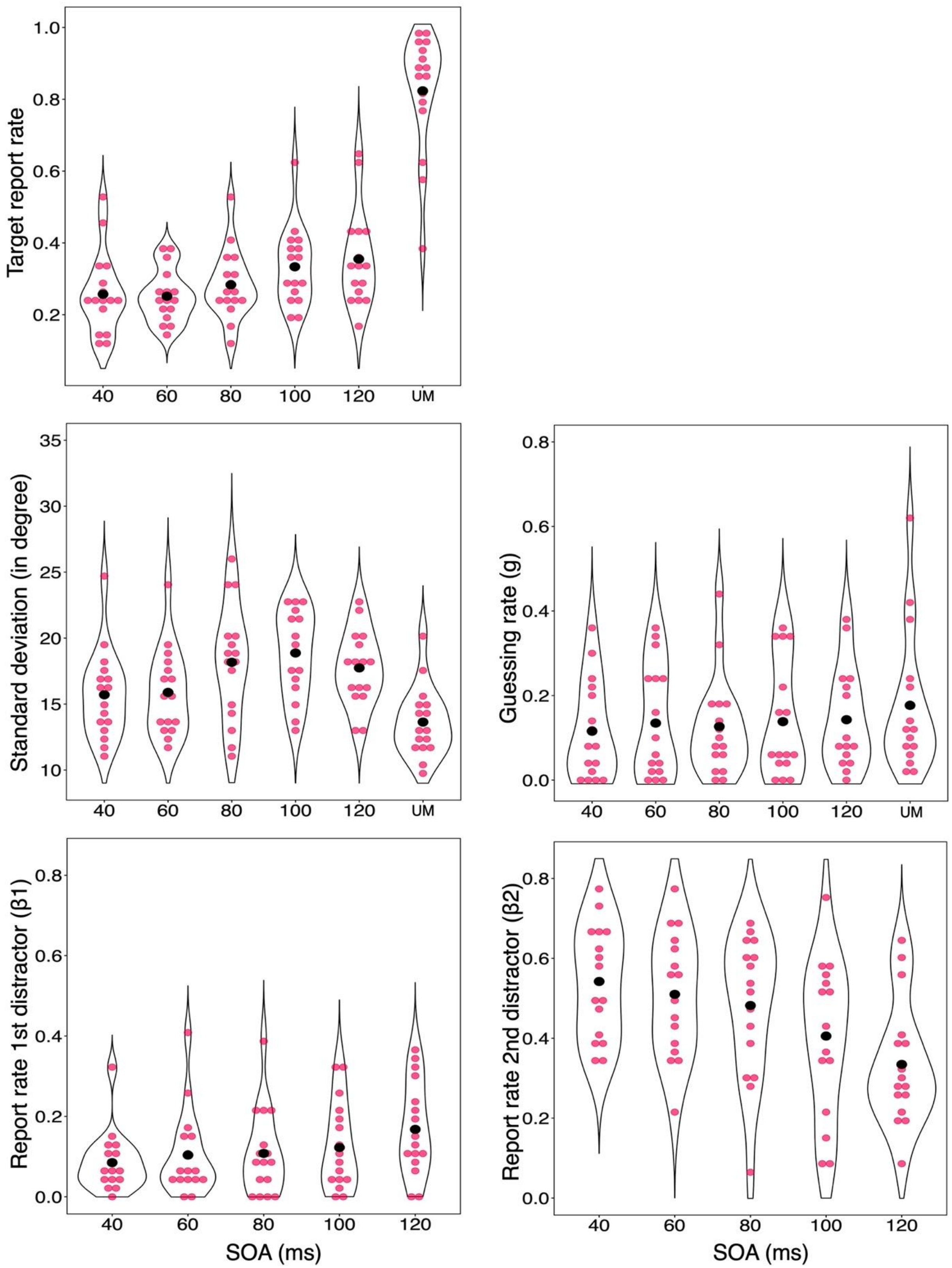

Supplementary Figure 6. Violin plots and dot plots (binned individual data in pink, average in black) of the mixture model parameters as a function of SOA in the masking condition and the unmasked (UM) condition of Experiment 3.
